# Delirium in Patients With Traumatic Brain Injury and Long‐Term Cognitive Decline: A Global Federated Real World Data Analysis

**DOI:** 10.1111/eci.70262

**Published:** 2026-10-03

**Authors:** Amirreza Sabahizadeh, Amir Askarinejad, Koohyar Ahmadzadeh, Seyed Reza Mirjalili, Khashayar Farshid, Leandra Serio, Enrico Tartaglia, Riccardo Proietti, Rhys Davies, Gregory Y. H. Lip

**Affiliations:** ^1^ Institute of Life Course and Medical Sciences University of Liverpool Liverpool UK; ^2^ Department of Life, Health & Environmental Sciences University of L'Aquila L'Aquila Italy; ^3^ Internal Medicine and Nephrology Division, ASL1 Avezzano‐Sulmona‐L'Aquila San Salvatore Hospital L'Aquila Italy; ^4^ Cardiology Division, Department of Biomedical Metabolic and Neural Sciences University of Modena and Reggio Emilia Modena Italy; ^5^ Clinical and Experimental Medicine PhD Programme University of Modena and Reggio Emilia Modena Italy; ^6^ Department of Cardiovascular and Metabolic Medicine, Institute of Life Course and Medical Sciences, Faculty of Health and Life Sciences University of Liverpool Liverpool UK; ^7^ The Walton Centre NHS Foundation Trust Liverpool UK; ^8^ Department of Clinical Medicine Aalborg University Aalborg Denmark; ^9^ Department of Cardiology, Lipidology and Internal Medicine Medical University of Bialystok Bialystok Poland

**Keywords:** cognitive outcomes, delirium, traumatic brain injury

## Abstract

**Background and Objective:**

Delirium may occur in patients with traumatic brain injury (TBI), but this may be a marker for subsequent cognitive decline post‐TBI. We aimed to compare cognitive outcomes and all‐cause mortality between TBI patients with and without delirium.

**Methods:**

We conducted a retrospective cohort study using the TriNetX global federated research network. We compared TBI patients who developed delirium within 14 days of TBI diagnosis with those who did not. The primary outcome was the 2‐year composite risk of dementia, vascular dementia and Alzheimer's disease. Secondary outcomes included dementia, vascular dementia, Alzheimer's disease and all‐cause mortality. Pre‐specified subgroup analyses were performed in female patients, older adults (≥ 75 years), and individuals with substance use.

**Results:**

TBI patients with delirium (*n* = 2564; mean age, 66.6 ± 19.4 years; 59.4% male), when compared with TBI patients without delirium (*n* = 667,950; mean age, 48.0 ± 25.1 years; 60.3% male), had more baseline comorbidities, including psychiatric disease, cerebrovascular disease and ischemic heart disease. After propensity score matching, TBI patients with delirium, compared with those without delirium, had a higher 2‐year risk of the primary composite outcome (HR, 2.47; 95% CI, 1.84–3.31), as well as dementia (HR, 2.63; 95% CI, 1.92–3.60), vascular dementia (HR, 2.76; 95% CI, 1.45–5.24) and Alzheimer's disease (HR, 2.23; 95% CI, 1.21–4.09).

**Conclusion:**

Delirium following TBI is associated with an increased risk of dementia and Alzheimer's disease. Delirium post‐TBI may be a marker or a contributing factor to long‐term cognitive decline in TBI patients.

## Graphical abstract

In this global federated TriNetX cohort, traumatic brain injury patients who developed delirium within 14 days were compared with those without delirium. After 1:1 propensity‐score matching, delirium was associated with higher two‐year risks of composite cognitive decline, dementia, vascular dementia, Alzheimer's disease and all‐cause death. The strongest association was observed for the composite cognitive outcome (HR 2.47; 95% CI 1.84–3.31). Delirium after traumatic brain injury may identify patients needing closer cognitive monitoring and follow‐up, particularly older adults at risk.

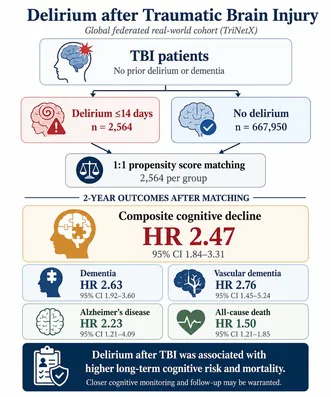

## 1 Introduction

Traumatic brain injury (TBI) results when external physical forces impact the head with sufficient intensity to cause damage to the brain [1]. TBI affects more than 50 million individuals annually and accounts for approximately 30%–40% of all injury‐related deaths worldwide [2, 3].

TBI is increasingly recognized as a chronic health condition with evolving neurological consequences that extend far beyond the acute injury period [4]. Emerging evidence demonstrates that TBI is associated with a substantially increased risk of neurodegenerative diseases, including Alzheimer's disease, Parkinson's disease, dementia with Lewy bodies and frontotemporal dementia [4, 5, 6, 7].

Delirium superimposed on dementia is highly prevalent among hospitalized older patients and is associated with significant adverse outcomes, including accelerated cognitive decline, increased risk of incident dementia, hospitalization and mortality [8]. Furthermore, cohort studies suggest that delirium itself is a strong predictor of subsequent dementia and death in older hospitalized populations [8, 9].

To the best of our knowledge, no prior study has directly compared cognitive outcomes between TBI patients with and without associated delirium. Therefore, we aimed to address this gap by comparing these two groups and evaluating the 2‐year risk of cognitive decline, including dementia, vascular dementia and Alzheimer's disease, as well as all‐cause mortality.

## 2 Methods

### 2.1 Study Design

This retrospective observational study was conducted using the TriNetX Research Network (TriNetX, Cambridge, MA, USA), a global federated health research network that provides access to anonymized electronic medical records from over 130 healthcare organizations, including academic medical centres, community hospitals and outpatient clinics. The network includes more than 300 million individuals, primarily in the United States (U.S.).

Available data include demographics, diagnoses coded using the International Classification of Diseases, Ninth and Tenth Revisions, Clinical Modification (ICD‐9‐CM and ICD‐10‐CM) codes and medications coded with Veteran Affairs (VA) codes or RxNorm classification systems, depending on the contributing institution. Further information about the network is available online (https://trinetx.com/data‐sets‐analytics/).

TriNetX operates in compliance with the Health Insurance Portability and Accountability Act (HIPAA) and US federal law. All patient data are de‐identified in accordance with HIPAA's Privacy Rule, and no patient‐level identifiers are accessible. Access to the data is governed through formal data‐sharing agreements with participating institutions. As a federated network using only de‐identified data, studies conducted within TriNetX do not require approval from institutional review boards or informed consent. Additional methodological details are provided in the Supporting Information.

### 2.2 Cohort Definition

The searches on the TriNetX online research platform were performed on March 23, 2026, using the Global Collaborative Network. At the time of data extraction, 168 healthcare organizations across the U.S. had contributed eligible patient data.

Based on recorded ICD‐10‐CM codes, two cohorts were constructed. The first cohort included patients with TBI who developed delirium within 14 days and had no prior history of delirium or dementia. This cohort comprised patients with a recorded diagnosis of TBI (codes S06.1–S06.9 and S06.A) between January 1, 2014, and January 1, 2024, along with a subsequent diagnosis of delirium (codes F05 or R41.0). The second cohort included patients with TBI who did not develop delirium within 14 days and had no prior history of delirium or dementia. This cohort comprised patients with a recorded diagnosis of TBI (codes S06.1–S06.9 and S06.A) between January 1, 2014, and January 1, 2024, with no recorded diagnosis of delirium (codes F05 or R41.0) during the study period. Patients with any documented history of delirium or dementia (codes F01–F03, G30, F05 or R41.0) prior to the TBI diagnosis were excluded. A detailed description of the ICD‐10‐CM codes, cohort eligibility criteria, exclusion criteria, and temporal definitions used for cohort construction is provided in Table S3.

### 2.3 Outcomes

The primary outcome was the two‐year risk of the composite outcome of cognitive decline (F01–F03 and G30). The secondary outcomes included the two‐year risk of dementia (F02, F03), vascular dementia (F01), Alzheimer's disease (G30) and all‐cause death. Follow‐up began on day 181 after the index event, with outcomes assessed from day 181 to day 730.

### 2.4 Statistical Analysis

Baseline characteristics of TBI patients with delirium versus TBI patients without delirium were compared and balanced using propensity‐score matching (PSM) at a 1:1 ratio. Matching was performed using the greedy nearest neighbour method, employing a caliper of 0.1.

The following covariates were included in the PSM model: age, sex, ethnicity, hypertension, cerebrovascular disease, diabetes mellitus, peripheral vascular disease, ischaemic heart disease, heart failure, mental and behavioural disorders due to psychoactive substance use, acute kidney failure, and chronic kidney disease, injuries to the head and surgical procedures on the skull, meninges and brain. The balance of demographic and clinical variables between groups was assessed using Absolute Standardized Mean Differences (ASD), with an ASD < 0.1 considered indicative of adequate balance.

Univariable Cox proportional hazards models, before and after PSM, were used to calculate hazard ratios (HRs) and 95% confidence intervals (95% CIs) for the risk of adverse events in TBI patients with delirium compared to TBI patients without delirium. Aalen–Johansen cumulative incidence curves were used to estimate the incidence of all‐cause death and the composite cognitive outcome, accounting for competing risks between these events.

Pre‐specified subgroup analyses were performed to explore potential effect modification. Subgroups were defined according to: (i) patient characteristics, including age (patients aged ≥ 75 years) and sex (female); and (ii) history of substance use (F10, F11, and F17). For each subgroup, analyses were conducted for both primary comparisons (TBI patients with delirium vs. TBI patients without delirium). For each subgroup comparison, separate propensity‐score matching, and survival analyses were performed using the same matching criteria and statistical methods described for the primary analysis.

All statistical analyses were conducted using the TriNetX analytics platform, which incorporates R (v3.2–3) and Python 3.7 libraries (scikit‐learn for logistic regression; survival for Cox models). Missing values were not imputed. All tests were two‐tailed, with statistical significance defined as *p* < 0.05.

## 3 Results

The final cohorts included 2564 TBI patients with delirium (mean age 66.6 ± 19.4 years; 59.4% male) 667,950 TBI patients without delirium (mean age 48.0 ± 25.1 years; 60.3% male) (Table 1). The study flow‐chart is reported in Figure 1.

**TABLE 1:** Baseline characteristics of the cohorts before and after propensity score matching.

	TBI patients with delirium versusTBI patients without delirium				
	Before PSM		After PSM		
	TBI patients with delirium (*N* = 2564)	TBI patients without delirium (*N* = 667,950)	TBI patients with delirium (*N* = 2564)	TBI patients without delirium (*N* = 2564)	ASD
Age at Index	66.6 ± 19.4	48.0 ± 25.1	66.6 ± 19.4	67.4 ± 19.1	0.042
Male	1523 (59.4%)	402,637 (60.3%)	1523 (59.4%)	1527 (59.6%)	0.003
White	1818 (70.9%)	369,918 (55.4%)	1818 (70.9%)	1804 (70.4%)	0.012
Black or African American	259 (10.1%)	77,388 (11.6%)	259 (10.1%)	228 (8.9%)	0.041
Not Hispanic or Latino	1882 (73.4%)	391,714 (58.6%)	1882 (73.4%)	1877 (73.2%)	0.004
Hypertensive diseases	1767 (68.9%)	136,933 (20.5%)	1767 (68.9%)	1791 (69.9%)	0.020
Ischemic heart diseases	826 (32.2%)	52,552 (7.9%)	826 (32.2%)	828 (32.3%)	0.002
Diabetes mellitus	752 (29.3%)	60,392 (9.0%)	752 (29.3%)	750 (29.3%)	0.002
Peripheral artery disease	656 (25.6%)	37,313 (5.6%)	656 (25.6%)	631 (24.6%)	0.022
Heart failure	543 (21.2%)	27,977 (4.2%)	543 (21.2%)	512 (20.0%)	0.030
Cerebrovascular diseases	1505 (58.7%)	66,927 (10.0%)	1505 (58.7%)	1480 (57.7%)	0.020
Mental and behavioural disorders due to psychoactive substance use	912 (35.6%)	84,609 (12.7%)	912 (35.6%)	890 (34.7%)	0.018
Acute kidney failure or chronic kidney disease	923 (36.0%)	43,620 (6.5%)	923 (36.0%)	909 (35.5%)	0.011
Surgical procedures on the skull, meninges and brain	237 (9.2%)	8545 (1.3%)	237 (9.2%)	210 (8.2%)	0.037

**FIGURE 1:**
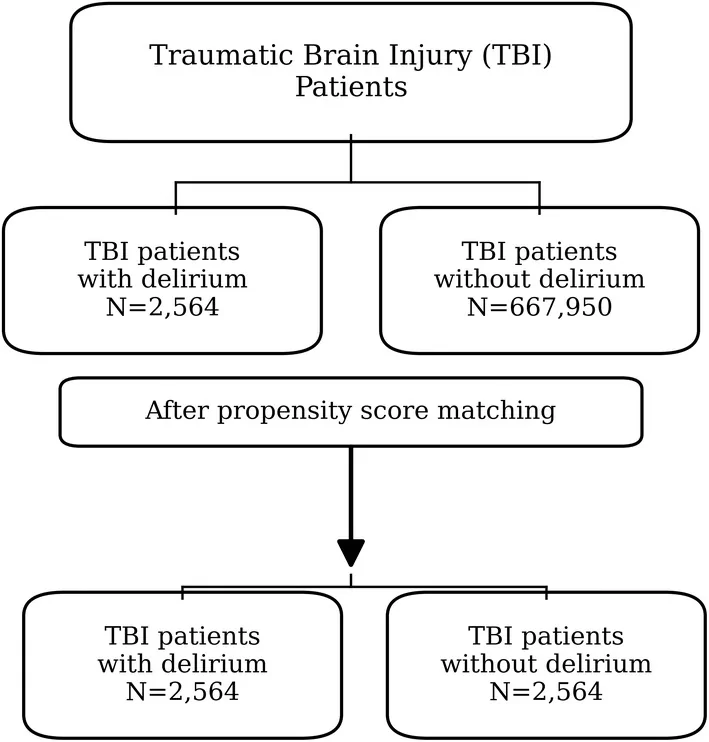
Study flowchart.

Before PSM, TBI patients with delirium were older and exhibited a higher burden of comorbidities compared with TBI patients without delirium (Table 1). For instance, hypertension, diabetes mellitus, ischaemic heart disease and heart failure were all more common among TBI patients with delirium compared with their counterparts (Table 1). Before PSM, when compared to TBI without delirium, TBI patients with delirium showed a higher risk of all‐cause death, dementia, vascular dementia, Alzheimer's disease and composite outcomes of cognitive decline (HR 6.63, 95% CI 5.60–7.86; Table 2, Figure S1).

**TABLE 2:** Cox Proportional‐Hazard regression of all‐cause death, composite outcome, dementia, vascular dementia and Alzheimer's disease in TBI patients with delirium versus TBI patients without delirium.

		Before PSM			After PSM		
		HR (95% CI)	Cohort I (*N* = 2564)	Cohort II (*N* = 667,950)	HR (95% CI)	Cohort I (*N* = 2564)	Cohort II (*N* = 2564)
TBI patients with delirium versus TBI patients without delirium	Composite outcome	6.63 (5.60, 7.86)	136 (4.7%)	6343 (0.9%)	2.47 (1.84, 3.31)	124 (4.8%)	71 (2.8%)
	Dementia	7.01 (5.85, 8.39)	121 (4.2%)	5337 (0.7%)	2.63 (1.92, 3.60)	111 (4.3%)	60 (2.3%)
	Vascular Dementia	9.14 (6.35, 13.14)	30 (1.0%)	988 (0.1%)	2.76 (1.45, 5.24)	28 (1.1%)	14 (0.55%)
	Alzheimer's disease	6.78 (4.69, 9.80)	29 (1.0%)	1289 (0.20%)	2.23 (1.21, 4.09)	27 (1.1%)	17 (0.66%)
	All‐cause death	3.65 (3.17, 4.20)	198 (6.8%)	16,518 (2.2%)	1.50 (1.21, 1.85)	181 (7.1%)	170 (6.6%)

Abbreviations: HR, hazard ratio; TBI, traumatic brain injury.

After PSM, baseline characteristics, including demographics, cardiovascular comorbidities and surgical procedures, were well balanced across groups, with all ASDs below 0.1 (Table 1). After PSM, TBI patients with delirium compared to TBI patients without delirium had a higher risk of all‐cause death (HR 1.50, 95% CI 1.21–1.85), dementia (HR 2.63, 95% CI 1.92–3.60), vascular dementia (HR 2.76, 95% CI 1.45–5.24), Alzheimer's disease (HR 2.23, 95% CI 1.21–4.09) and composite outcomes of cognitive decline (HR 2.47, 95% CI 1.84–3.31) (Table 2, Figure S2).

### 3.1 Multivariable Cox Proportional‐Hazards Model

In the multivariable Cox proportional‐hazards model, age (HR 1.07, 95% CI 1.072–1.077), hypertensive disease (HR 1.12, 95% CI 1.06–1.20), diabetes mellitus (HR 1.29, 95% CI 1.20–1.38), major depressive disorder (HR 1.41, 95% CI 1.23–1.61), cerebral infarction (HR 1.58, 95% CI 1.45–1.72) and Parkinson's disease (HR 2.63, 95% CI 2.31–3.00) were significantly associated with the composite outcome (Table 3).

**TABLE 3:** Multivariable Cox proportional hazards model for predictors of incident composite outcome.

Predictor	HR (95% CI)
Age at index	1.075 (1.072–1.077)
Male sex	0.91 (0.86–0.96)
Hypertensive disease	1.12 (1.06–1.20)
Diabetes mellitus	1.29 (1.20–1.38)
Major depressive disorder	1.41 (1.23–1.61)
Cerebral infarction	1.58 (1.45–1.72)
Parkinson's disease	2.63 (2.31–3.00)

Abbreviations: CI, confidence interval; HR, hazard ratio.

### 3.2 Subgroup Analyses

After PSM, in the elderly age subgroup (≥ 75 years), the pattern of results mirrored the primary analysis. TBI patients with delirium showed a higher risk of developing the composite dementia outcome within 2 years compared with TBI patients without delirium (HR 1.70, 95% CI 1.17–2.47) (Figure 2).

**FIGURE 2:**
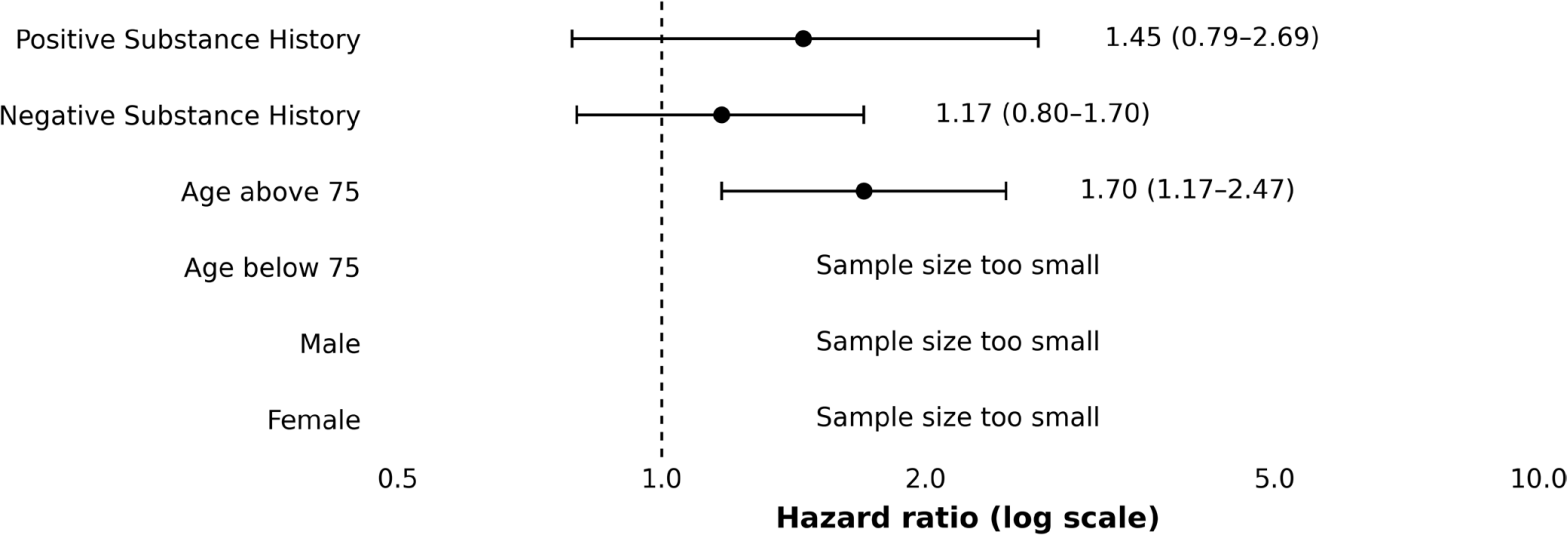
After PSM hazard ratios for subgroups comparing the 2‐year risk of composite cognitive outcome, including dementia, vascular dementia and Alzheimer's disease—between TBI patients with and without delirium.

### 3.3 Sensitivity Analyses

In a sensitivity analysis restricting the follow‐up period to 6–18 months, the results remained consistent with the primary analysis. Compared with TBI patients without delirium, TBI patients with delirium had a higher risk of the composite outcome both before propensity‐score matching (HR 5.06, 95% CI 3.90–6.58) and after matching (HR 1.80, 95% CI 1.19–2.72) (Table S1).

In a second sensitivity analysis excluding patients who died between 1 January 2014 and 1 January 2024, the association remained significant. TBI patients with delirium had a higher risk of the composite outcome compared with TBI patients without delirium, both before PSM (HR 7.12, 95% CI 5.82–8.69) and after PSM (HR 2.64, 95% CI 1.85–3.78; Table S2).

### 3.4 Cumulative Incidence of Outcomes

Among TBI patients with delirium, 174 patients (6.79%) died, with an estimated cumulative incidence of 11.39%, while 134 patients (5.23%) developed the composite dementia outcome, with an estimated cumulative incidence of 8.62% (Figure S3).

Among TBI patients without delirium, 15,119 patients (2.26%) died, with an estimated cumulative incidence of 3.51%, while 6228 patients (0.93%) developed the composite dementia outcome, with an estimated cumulative incidence of 1.44% (Figure S4).

## 4 Discussion

In this study, our principal findings are as follows: (i) comorbidities including hypertensive disease, cerebrovascular disease and DM were more prevalent among TBI patients with delirium compared with those without delirium; notably, hypertension and diabetes mellitus were also independent predictors of the composite cognitive decline outcome; (ii) compared with TBI patients without delirium, those with delirium had a higher risk of developing dementia, vascular dementia and Alzheimer's disease individually, as well as the composite cognitive outcome comprising these conditions; (iii) TBI patients with delirium had a higher risk of 2‐year all‐cause mortality compared with TBI patients without delirium; (iv) in the subgroup analysis, delirium was associated with an increased risk of the composite cognitive decline outcome among TBI patients aged > 75 years.

TBI patients with delirium, compared with TBI patients without delirium, in our cohort were older and carried a greater burden of comorbidities including hypertension and diabetes mellitus. Delirium is associated with increased inflammation and greater vulnerability to cerebral insults, including hypoperfusion and hypertension, during a hospital stay [10, 11, 12]. Also, a meta‐analysis of 52 studies showed that patients with diabetes had approximately a 78% higher odds of developing delirium compared to non‐diabetic patients [13]. Consistent with our findings that hypertension and diabetes were independent predictors of the composite outcome, other studies have similarly shown that both hypertension and diabetes mellitus are independently associated with a higher risk of dementia [14, 15, 16]. Taken together, these findings suggest that comorbidities are common among patients with delirium and that appropriate management may help reduce the risk of subsequent cognitive decline.

TBI patients with delirium showed a higher risk of developing dementia within the 2‐year follow‐up compared with the non‐delirium group. It is well established that patients with TBI are at a substantial risk of developing both delirium in the acute phase and subsequent dementia. Delirium is particularly common among critically ill TBI patients, especially in intensive care settings, and is associated with adverse short‐term outcomes [17]. Moreover, growing evidence suggests that TBI confers an increased long‐term risk of cognitive decline and dementia, highlighting its role as an important contributor to chronic neurocognitive impairment. Additionally, recent studies have demonstrated a link between delirium episodes and the future development of dementia [18, 19, 20]. Hence, the development of delirium after TBI could be considered a marker of increased dementia risk. Therefore, close monitoring and more structured follow‐up may play an important role in slowing dementia progression in these high‐risk patients.

In our study, the risk of vascular dementia over the 2‐year follow‐up was higher in TBI patients with delirium than in those without delirium. Both TBI and delirium are significantly associated with an increased risk of vascular dementia [21, 22]. Notably, a systematic assessment reported that beta‐blockers and vasodilator antihypertensives were associated with a reduced risk of major neurocognitive disorders, including vascular dementia [23]. As comorbidities such as hypertension were more common in the delirium group, close monitoring and appropriate management of hypertension may play an important role in reducing the risk of future vascular dementia by preserving cerebrovascular integrity and limiting cumulative brain injury [24, 25].

Similar to other forms of dementia, TBI patients with delirium showed a higher risk of developing Alzheimer's disease within 2 years compared with the TBI patients without delirium. TBI, including mild cases, is associated with a modestly increased risk of Alzheimer's disease (RR = 1.18, 95% CI 1.11–1.25) [26]. In addition, delirium episodes significantly increased the risk of developing Alzheimer's disease [9]. Notably, the use of a combination of angiotensin‐converting enzyme inhibitors (ACEIs) and statins after TBI has been associated with a lower risk of possible Alzheimer's disease compared with no treatment [27]. Taken together, screening TBI patients with delirium, ensuring close follow‐up, and managing underlying comorbidities may play an important role in reducing the future risk of Alzheimer's disease.

TBI patients with delirium also had a higher risk of all‐cause mortality compared with the TBI patients without delirium. One recent meta‐analysis of 253 studies reported that patients who experienced delirium had increased mortality across all follow‐up periods after hospital discharge compared with those without delirium [28]. These findings are further supported by evidence that persistent delirium is associated with increased mortality, with the risk rising for each additional day of delirium, highlighting the importance of early detection and management [29]. In line with our findings, monitoring and follow‐up of patients with delirium episodes may play an important role in improving long‐term survival.

After PSM, our subgroup analysis of patients aged over 75 years confirmed the main findings. Age was an independent predictor of cognitive decline. Similarly, two studies in populations aged over 65 and 85 years reported that delirium was associated with higher rates of cognitive decline [9, 30]. Notably, a recent population‐based cohort study including 132,113 matched pairs of community‐dwelling adults aged over 65 years reported that late‐life TBI was associated with a 69% higher risk of incident dementia within 5 years [31]. In line with these findings, greater emphasis should be placed on monitoring and closer follow‐up in older TBI patients who develop delirium.

Given our findings, several important clinical implications should be considered. Early identification of delirium in patients with TBI may help identify individuals at higher risk of subsequent cognitive decline and mortality. This highlights the importance of implementing routine screening strategies, optimizing the management of comorbidities, and ensuring close follow‐up, particularly in older patients. In addition, targeted interventions aimed at reducing delirium duration and severity may have the potential to improve both cognitive and survival outcomes. Further prospective studies are needed to determine whether early and structured management of delirium can modify long‐term neurocognitive trajectories in this high‐risk population.

## 5 Limitations

This study has several limitations that should be considered. First, the diagnoses of delirium, TBI and dementia were based on ICD‐10 coding, which may be subject to misclassification or coding inaccuracies. Second, due to the observational and retrospective nature of the study, causality cannot be established, and only associations can be inferred. Third, despite propensity‐score matching and adjustment for multiple covariates, residual confounding from unmeasured variables may still be present. Fourth, selection bias may exist due to the inclusion of patients captured within the TriNetX network, which may not be fully representative of the general population. Finally, the retrospective design limits the ability to account for changes in clinical status, severity of TBI, or delirium duration, which may influence the observed outcomes.

## 6 Conclusion

In this large real‐world cohort study, delirium following TBI is associated with an increased risk of dementia, vascular dementia and Alzheimer's disease, as well as higher all‐cause mortality. Delirium post‐TBI may be a marker or a contributing factor to long‐term cognitive decline in TBI patients. Careful monitoring of patients with TBI and delirium, particularly older adults, might have a role in facilitating improved screening and timely referral to specialist services for cognitive decline assessment and work‐up.

## Author Contributions

**Amirreza Sabahizadeh:** visualization, writing – original draft, writing – review and editing; **Amir Askarinejad:** supervision, conceptualisation, writing – review and editing; **Koohyar Ahmadzadeh:** writing – original draft; **Seyed Reza Mirjalili:** review and editing; **Khashayar Farshid:** writing – original draft; **Leandra Serio:** formal analysis; **Enrico Tartaglia:** methodology; **Riccardo Proietti:** supervision; **Rhys Davies:** supervision; **Gregory Y. H. Lip:** conceptualisation, project administration, writing – review and editing and supervision.

## Funding

The authors have nothing to report.

## Ethics Statement

This study was conducted using the TriNetX Research Network, a global federated database of de‐identified electronic health records. TriNetX complies with the Health Insurance Portability and Accountability Act (HIPAA) Privacy Rule and applicable US federal regulations governing the use of de‐identified health data. As only anonymized, de‐identified data were used, institutional review board approval and informed consent were not required in accordance with applicable regulations and TriNetX policies.

## Conflicts of Interest

G.Y.H.L. is a consultant and speaker for BMS/Pfizer, Boehringer Ingelheim, Daiichi‐Sankyo, Anthos, Huawei, Skylabs.

## Supporting information


**Table S1:** Multivariable Cox proportional‐hazards regression of all‐cause death and composite cognitive outcomes with follow‐up limited to 6–18 months in TBI patients with delirium versus TBI patients without delirium.
**Table S2:** Multivariable Cox proportional‐hazards regression of composite cognitive outcomes after excluding deceased patients in TBI patients with delirium versus TBI patients without delirium.
**Table S3:** ICD‐10‐CM phenotype definitions, eligibility criteria and study timelines.
**Figure S1:** Hazard ratios comparing outcomes between TBI patients with delirium and TBI patients without delirium before PSM.
**Figure S2:** Hazard ratios comparing outcomes between TBI patients with delirium and TBI patients without delirium after PSM.
**Figure S3:** Aalen–Johansen cumulative incidence curves for TBI patients with delirium: purple indicates death, and green indicates the composite cognitive outcome.
**Figure S4:** Aalen–Johansen cumulative incidence curves for TBI patients without delirium: purple indicates death, and green indicates the composite cognitive outcome.

## Data Availability

Data used in this research project will be available upon a reasonable request from the corresponding authors.
