# Supplementary material for: Delirium in Patients With Traumatic Brain Injury and Long‐Term Cognitive Decline: A Global Federated Real World Data Analysis
Source: Eur J Clin Invest. 2026 Oct 3;56(10):e70262. doi: 10.1111/eci.70262 (PMC13633596; doi:10.1111/eci.70262)
Supplement: Supplementary file 1 [file ECI-56-e70262-s001.docx]

**Supplementary materials**

**Association Between Delirium and Cognitive Decline in Patients with Traumatic Brain Injury: A Global Federated Real-World Data Analysis**

**TriNetX Database**
The TriNetX data are collected from member healthcare organizations (HCOs) and derived from their primary electronic health record (EHR) systems. A typical HCO is a large academic medical centre, with data obtained from most of its affiliated facilities. A single HCO may include multiple sites, such as main and satellite hospitals. The data are stored within the TriNetX database via a physical server located in the institution’s data centre or through a virtual hosted appliance. The TriNetX platform consists of a network of these appliances connected in a federated system. This network enables queries to be broadcast to each appliance, after which the results are aggregated and returned.

Once data are received by the network, they are mapped to a standardized and controlled set of clinical terminologies and subjected to data quality assessments, including data cleaning processes that exclude records not meeting TriNetX quality standards. The TriNetX database performs continuous and extensive data quality checks with each update, assessing conformance, completeness, and plausibility (<http://doi.org/10.13063/2327-9214.1244>). Compliance with the Health Insurance Portability and Accountability Act (HIPAA) is ensured through data de-identification. Available data within the network include demographics, diagnoses (coded using ICD-10-CM), procedures (coded using ICD-10-PCS or CPT), and measurements (coded using LOINC).

While the dataset provides detailed information on diagnoses and procedures, other variables—such as socioeconomic and lifelong factors—are not comprehensively captured. A key advantage of EHR data compared with insurance claims data is that it includes both insured and uninsured individuals. In comparison with survey data, EHR data better reflect diagnostic rates among patients presenting to healthcare services, thereby providing a more accurate estimate of disease burden within healthcare systems.

A major limitation of relying on diagnosis codes is that they do not capture undiagnosed individuals who may have an existing condition but have not yet accessed medical care. Another limitation of EHR data is that patients may receive care across different HCOs, and if a given institution is not part of the federated network, portions of their medical records may be unavailable. Although the use of a network of HCOs, rather than a single site, reduces this issue, it does not eliminate it entirely.

**Propensity Score Matched Analyses**
Propensity score matched analyses were conducted using logistic regression (Logistic Regression from the scikit-learn package in Python, version 3.7). TriNetX implemented a 1:1 greedy nearest-neighbour matching approach with a caliper of 0.1 pooled standard deviations. To reduce potential bias associated with the nearest-neighbour algorithm, the data were randomized. Baseline characteristics with a standardized mean difference below 0.1 between groups were considered well balanced.

Assessment of the Proportional Hazards Assumption
To assess whether the proportional hazards assumption was met in the Cox regression models, we performed a Chi-square (χ²) test based on Schoenfeld residuals. These tests evaluate whether the relationship between covariates and the hazard function remains constant over time. The null hypothesis assumes that the effect of OAC discontinuation on the hazard of the primary outcomes is constant throughout the study period. The χ² statistic quantifies the deviation between observed and expected Schoenfeld residuals. A higher χ² value indicates greater deviation from expected values, suggesting a possible violation of the proportional hazards assumption, whereas a lower χ² value suggests closer agreement and supports the assumption.

The p-value, derived from the χ² statistic, indicates the probability of observing such deviations under the null hypothesis. A p-value greater than 0.05 suggests that any observed variation is likely due to chance, supporting the validity of the proportional hazards assumption. In contrast, a p-value below 0.05 indicates that the observed deviations are unlikely to be due to random variation, suggesting a violation of the proportional hazards assumption.

**Supplementary Table 1. Multivariable Cox Proportional-Hazards Regression of All-Cause Death and Composite Cognitive Outcomes with Follow-up Limited to 6–18 Months in TBI Patients with Delirium vs TBI Patients without Delirium**

|  | | **Before PSM** | | | **After PSM** | | |
| --- | --- | --- | --- | --- | --- | --- | --- |
|  |  | HR (95%CI) | Cohort I  (N=2,914) | Cohort II  (N=730, 715) | HR (95%CI) | Cohort I  (N=2,623) | Cohort II  (N=2,623) |
| **TBI patients with delirium vs TBI patients without delirium** | **Composite outcome** | 5.06 (3.90, 6.58) | 111 (3.8%) | 4,666 (0.6%) | 1.80 (1.19, 2.72) | 102 (3.9%) | 57 (2.2%) |
|  | **All-cause death** | 3.92 (3.34, 4.61) | 150 (5.1%) | 11,707 (1.6%) | 1.50 (1.21, 1.85) | 142 (5.4%) | 106 (4.0%) |

**Supplementary Table2. Multivariable Cox Proportional-Hazards Regression of Composite Cognitive Outcomes After Excluding Deceased Patients in TBI Patients with Delirium vs TBI Patients without Delirium**

|  | | **Before PSM** | | | **After PSM** | | |
| --- | --- | --- | --- | --- | --- | --- | --- |
|  |  | HR (95%CI) | Cohort I  (N=2,018) | Cohort II  (N=646,186) | HR (95%CI) | Cohort I  (N=1,772) | Cohort II  (N=1,772) |
| **TBI patients with delirium vs TBI patients without delirium** | **Composite outcome** | 7.12 (5.82, 8.69) | 98 (4.9%) | 4,869 (0.8%) | 2.64 (1.85, 3.78) | 91 (5.1%) | 45 (2.5%) |

**Supplementary Table 3. ICD-10-CM Phenotype Definitions, Eligibility Criteria, and Study Timelines**

**Index date:** the date of the qualifying traumatic brain injury (TBI) diagnosis. Diagnostic phenotypes were constructed from routinely recorded ICD-10-CM codes in the TriNetX electronic health-record network. The table below distinguishes the pre-index exclusion period, the 14-day post-TBI exposure-classification window, and the subsequent outcome-assessment window.

| **Domain / purpose** | **ICD-10-CM code(s)** | **Phenotype / code concept** | **Operational definition** | **Time window relative to TBI index** | **Role in analysis** |
| --- | --- | --- | --- | --- | --- |
| Index condition: TBI | S06.1-S06.9; S06.A | Intracranial injury / traumatic brain injury code families | A qualifying TBI diagnosis recorded between 1 January 2014 and 1 January 2024 defined the index event. | Day 0 (index date) | Required for both cohorts |
| Exposure: post-TBI delirium | F05; R41.0 | F05: delirium due to known physiological condition; R41.0: disorientation, unspecified | Patients were classified in the TBI-with-delirium cohort when a new qualifying delirium/disorientation code was recorded after the index TBI and within the prespecified acute exposure window. | Day 0 through Day 14 after index TBI | Defines exposed cohort |
| Comparator: no early post-TBI delirium | Absence of F05 or R41.0 in exposure window | No qualifying delirium/disorientation code during the acute post-TBI classification window | Patients with TBI who did not have a qualifying F05/R41.0 record during the first 14 days after the index TBI were classified as the comparator cohort, provided the pre-index exclusion criteria were also met. | Day 0 through Day 14 after index TBI | Defines unexposed comparator cohort |
| Pre-index exclusion: prior delirium | F05; R41.0 | Prior delirium/disorientation | Any patient with a qualifying delirium/disorientation code recorded before the index TBI was excluded so that the delirium exposure represented a new post-TBI event rather than pre-existing delirium. | All available history before Day 0 (database inception/earliest available record to index date) | Exclusion criterion for both cohorts |
| Pre-index exclusion: prior dementia / Alzheimer disease | F01-F03; G30 | F01: vascular dementia; F02: dementia in other diseases classified elsewhere; F03: unspecified dementia; G30: Alzheimer disease | Any patient with a qualifying dementia or Alzheimer disease code recorded before the index TBI was excluded to reduce inclusion of patients with established cognitive disease at baseline. | All available history before Day 0 (database inception/earliest available record to index date) | Exclusion criterion for both cohorts |
| Primary outcome: composite cognitive outcome | F01-F03; G30 | Composite of vascular dementia, other/unspecified dementia, and Alzheimer disease | First recorded qualifying diagnosis during follow-up contributed to the composite cognitive outcome. | Day 181 through Day 730 after index TBI | Primary outcome |
| Secondary outcome: dementia | F02; F03 | Dementia in other diseases classified elsewhere; unspecified dementia | First recorded qualifying dementia diagnosis during follow-up. | Day 181 through Day 730 after index TBI | Secondary outcome |
| Secondary outcome: vascular dementia | F01 | Vascular dementia | First recorded qualifying vascular dementia diagnosis during follow-up. | Day 181 through Day 730 after index TBI | Secondary outcome |
| Secondary outcome: Alzheimer disease | G30 | Alzheimer disease | First recorded qualifying Alzheimer disease diagnosis during follow-up. | Day 181 through Day 730 after index TBI | Secondary outcome |
| Secondary outcome: all-cause death | Not ICD-10-CM phenotype | Death recorded in TriNetX | All-cause mortality assessed during the same outcome follow-up period. | Day 181 through Day 730 after index TBI | Secondary outcome |
| Sensitivity analysis: alternative outcome window | Same cognitive outcome codes as above | Composite cognitive outcome | The cognitive outcome analysis was repeated using a shorter follow-up interval to assess robustness to the choice of follow-up window. | 6 to 18 months after index TBI | Sensitivity analysis |

**Figure 1. Hazard ratios comparing outcomes between TBI patients with delirium and TBI patients without delirium before PSM**

**Figure 2. Hazard ratios comparing outcomes between TBI patients with delirium and TBI patients without delirium after PSM**

**Figure 3. Aalen–Johansen cumulative incidence curves for TBI patients with delirium: purple indicates death, and green indicates the composite cognitive outcome.**

**Figure 4. Aalen–Johansen cumulative incidence curves for TBI patients without delirium: purple indicates death, and green indicates the composite cognitive outcome.**
